# Urethral reconstructive surgery: Which catheters are better?

**Published:** 2008

**Authors:** Manav Suryavanshi, Rajeev Kumar

**Affiliations:** Department of Urology, All India Institute of Medical Sciences, New Delhi - 110 029, India. E-mail: rajeev02@gmail.com

## SUMMARY

This is a prospective randomized study of 85 men undergoing urethral reconstruction from February 2004 to August 2006, as performed by one surgeon (CMG). Randomization was achieved by alternating the use of latex based (42) or all-silicone (43) catheters after each respective case unless there were concerns about latex sensitivity. Median follow-up was 20 months (range 10-36). Mean patient age, urethral stricture length, stricture etiology, specific type of reconstructive procedure, and complication rate for each type of repair did not significantly differ between the two groups. No patients in the latex group experienced a problem with latex sensitivity and there were no catheter-related incidents in either group. Stricture length was the only factor appearing to affect stricture recurrence, although statistical significance was not seen (6.7 vs. 5.4 cm, *P*= 0.32). There was no difference with regard to catheter type (*P*= 0.97). Median time to stricture recurrence was not statistically different between the silicone and latex groups (85.1 vs. 112.5 days, range 37-219, *P*= 0.83).

## COMMENTS

Latex catheters without a protective coating generate periurethral inflammatory reaction.[1] Coated latex catheters claiming to be as safe as silicone catheters were developed to prevent these complications, were less expensive and, being less stiff, were more comfortable. These catheters are now used in more than 90% of patients requiring urethral catheterization. Silicone is entirely biocompatible and generates little urethral epithelial inflammation, minimizing scar formation, and suture line contracture after urethroplasty.[2] The increased strength of silicone catheters makes them stiff, causing more discomfort. The hydrogel coated latex-based Foley catheters absorbs water, forming a thin aqueous film outside the catheter increasing smoothness, lubricity, and protecting the urethra from the underlying latex.[2] However, problems with hydrogel coated latex catheters include nonuniform hydrogel coating and cracking of the hydrogel surface after catheter bending illustrating that even with the protective coating there remains the potential for the urethral epithelium and new anastomosis to be exposed to raw latex material.[3] One of the areas where such exposure may be of significant concern is in urethroplasties. As such, restructuring and fistulae are significant complications of such surgeries and most surgeons are fastidious about their choice of catheters to splint the anastomosis. However, the authors in this study found no statistical differences in terms of the complication or recurrence rate between the two catheters when used after urethral reconstruction. The study was underpowered to detect minor differences between the catheters and does not conclude that one type of catheter is superior to the other. Nonetheless, the results emphasize that the only requirements for urethral catheters after urethral reconstructive surgery are to be biocompatible, inert and to drain the bladder adequately.
